# Manipulation of Superparamagnetic Beads on Patterned Exchange-Bias Layer Systems for Biosensing Applications

**DOI:** 10.3390/s151128854

**Published:** 2015-11-13

**Authors:** Arno Ehresmann, Iris Koch, Dennis Holzinger

**Affiliations:** Institute of Physics and Center for Interdisciplinary Nanostructure Science and Technology (CINSaT), University of Kassel, Heinrich-Plett-Str.40, Kassel D-34132, Germany; E-Mails: ehresmann@physik.uni-kassel.de (A.E.); iris.koch@physik.uni-kassel.de (I.K.)

**Keywords:** exchange-bias, ion bombardment induced magnetic patterning (IBMP), magnetic stray fields, magnetic field landscape design, superparamagnetic bead, particle transport, potential energy landscape, biosensor, DOWMAT, microfluid mixing, scanning Hall probe microscopy

## Abstract

A technology platform based on a remotely controlled and stepwise transport of an array arrangement of superparamagnetic beads (SPB) for efficient molecular uptake, delivery and accumulation in the context of highly specific and sensitive analyte molecule detection for the application in lab-on-a-chip devices is presented. The near-surface transport of SPBs is realized via the dynamic transformation of the SPBs’ magnetic potential energy landscape above a magnetically stripe patterned Exchange-Bias (EB) thin film layer systems due to the application of sub-mT external magnetic field pulses. In this concept, the SPB velocity is dramatically influenced by the magnitude and gradient of the magnetic field landscape (MFL) above the magnetically stripe patterned EB substrate, the SPB to substrate distance, the magnetic properties of both the SPBs and the EB layer system, respectively, as well as by the properties of the external magnetic field pulses and the surrounding fluid. The focus of this review is laid on the specific MFL design in EB layer systems via light-ion bombardment induced magnetic patterning (IBMP). A numerical approach is introduced for the theoretical description of the MFL in comparison to experimental characterization via scanning Hall probe microscopy. The SPB transport mechanism will be outlined in terms of the dynamic interplay between the EB substrate’s MFL and the pulse scheme of the external magnetic field.

## 1. Introduction

The advent of micro-total-analysis-systems (µTAS) and lab-on-a-chip (LOC) devices will enable the transfer of conventional (bio)chemical analysis techniques, usually carried out by expensive and bulky laboratory equipment, to handheld and comparably inexpensive devices on centimeter length scales [1,2,3,4,5,6,7,8]. As biochemical analysis procedures are naturally carried out in a liquid or physiological environment, the µTAS and LOC devices must be able to perform their tasks when the molecules or cells of interest are dispersed or dissolved in a liquid [9]. The liquid volume being processed within a certain timeframe in the chip is therefore one out of several boundary conditions which is crucial for all possible chip functionalities. If, e.g., the concentration of a certain biomolecule in a liquid shall be determined (henceforth referred to as the analyte) and the analyte is expected to be present at a low concentration, say in the femtomolar range (*i.e.*, 6 × 10^8^ molecules per liter), a volume of 1 µL (1 mm^3^) solvent contains only about 600 molecules, an extremely challenging task for detection [10]. This is even more severe when considering the fact that the usual physiological liquids, like blood serum or urine, not only contain the analyte molecules, but also a manifold of other molecules usually present at much higher concentrations [10]. Such other molecules may result in additional detected signals, which might interfere with the detection of the analyte [10]. If analyte capture molecules, e.g., natural or synthetic antibodies, are employed in the µTAS or LOC device for analyte detection, this already imposes severe requirements for the choice of capture molecules: the capture molecule must have a high binding rate for quick binding and the binding to the analyte must be highly specific [10]. If, moreover, the process time for the chip operation is set, the dissociation timescale of the capture-molecule-analyte-complex must be sufficiently large to ensure that the capture-molecule-analyte-complex is kinetically stable during the chip operation time.

Another requirement for the design of such an LOC system needs a more fundamental discussion on the detection of analyte molecules in terms of physical quantities. Independent of the detection method used to quantify the analyte molecules (e.g., fluorescence detection (FD), magnetoresistive detection (MD), reflected electromagnetic radiation for surface plasmon resonance displacement detection (SPR)) [6,11,12,13,14,15,16,17,18,19,20,21,22,23,24,25], the detector read out signal is always related to a density of the physical quantity to be measured. For FD this is the fluorescence intensity impinging on the detector surface which is proportional to the emitted fluorescence intensity of the source volume, for MD this is the magnetic flux density of analyte loaded superparamagnetic beads immobilized above the sensor area, and for SPR this is the change in the optical refraction index averaged over the volume close to the metal layers due to the binding of analyte molecules to immobilized capture molecules above an organic matrix. Accordingly, for a given detection limit of a detector the density of the measured physical quantity is an important feature. The second requirement for the layout of an LOC system is therefore to maximize the density of the measured physical quantity while simultaneously considering the desired dynamic range for detection [26]. At the same time, noise and unspecific background signals have to be minimized to maximize the signal-to-noise ratio [10].

The given detection limit of the measurement system requires a minimum density of the measured physical quantity and therefore a minimum density of analyte molecules within the detection volume. Since the detection volume is usually smaller in comparison to the liquid volume of the whole µTAS or LOC device, the naturally occurring number of analyte molecules within the detection volume may not be sufficient in order to generate a significant measuring signal. In this case, an efficient and specific transport of analyte molecules from the available liquid into the investigated detection volume is necessary [27]. If the number of analyte molecules within the whole chip volume is still too small to be detected, the investigated liquid has to be exchanged. Therefore, the necessary fluid volume has to be transported through the micro channels of the chip, which requires microfluidic channels associated with syringe pumps together with a capture and concentration technology for the analyte molecules on chip. Finally, for efficient analyte detection an analyte-specific and sensitive quantitative detection technology must be available.

Although in principle such devices can be realized using a combination of a fluid flow in microfluidic channels together with capture molecule-functionalized surfaces or superparamagnetic beads for the transport of biomolecules or cells [28,29,30,31], several technical and practical challenges have to be met, covering both the type of beads and the transport concept to be used as well as the class of capture molecules. Here, we report on a technology platform based on a remotely controlled transport of capture molecule functionalized superparamagnetic beads (SPBs) for efficient molecular uptake, transport, and concentration for sensitive detection of analyte molecules [32]. The focus is laid on the fundamental physics and the technology of the SPB transport concept rather than on the challenges associated with the biochemistry of the capture molecules and their attachment to the SPBs, the analyte binding and dissociation process as well as the specificity or the binding of analyte molecules to chip surfaces.

The central feature of this technology platform is the use of SPBs transported above the chip surface by applying weak external magnetic field pulses of only a few millitesla [27]. The SPBs are used to perform several tasks of the LOC device, which will be outlined below and which will essentially fulfill the above mentioned requirements for a state-of-the-art LOC device for sensitive analyte molecule detection.

SPBs may be transported by magnetic gradient fields. The magnetic force ${\vec{F}}_{M}$ acting on a SPB (hydrodynamic volume $V_{SPB}$, volume averaged susceptibility $\chi_{SPB}$) in a fluid (volume averaged susceptibility $\chi_{L}$) induced by a magnetic gradient field $\vec{B}$ can be written as [33,34]:

(1) $${\vec{F}}_{M}=\frac{V_{SPB}\cdot \left(\chi_{SPB}-\chi_{L}\right)}{{µ}_{0}}(\vec{B}\cdot \nabla )\vec{B}$$

Although SPB movement by this force in liquids is well-known and frequently applied in biotechnology, it poses considerable technical challenges when trying to apply it in LOC devices. For SPBs moving in solution while exposed to an external magnetic gradient field, particle agglomeration occurs due to the attractive long-range magnetostatic interaction between neighboring SPBs parallel to the direction of the magnetic field lines [33,35]. Moreover, the experimentally observed bead velocities are comparably small (in the range of a few µm/s) when both strong magnetic fields and gradients are applied [26,35]. Therefore, strong laboratory magnetic field sources are required for the practical use of conventional magnetophoresis in LOC devices [26]. Even if such strong magnetic field sources are used, transport times of several hours are still necessary to achieve a transport of SPBs over centimeter distances [26]. To overcome these drawbacks, static magnetic stray field landscapes (MFLs), locally varying on the micrometer scale, overlaid by comparably weak dynamically varying (homogeneous) external magnetic fields were introduced as a novel technique to transport SPBs. This idea has boosted the field with a variety of technological realizations.

**Figure 1:**
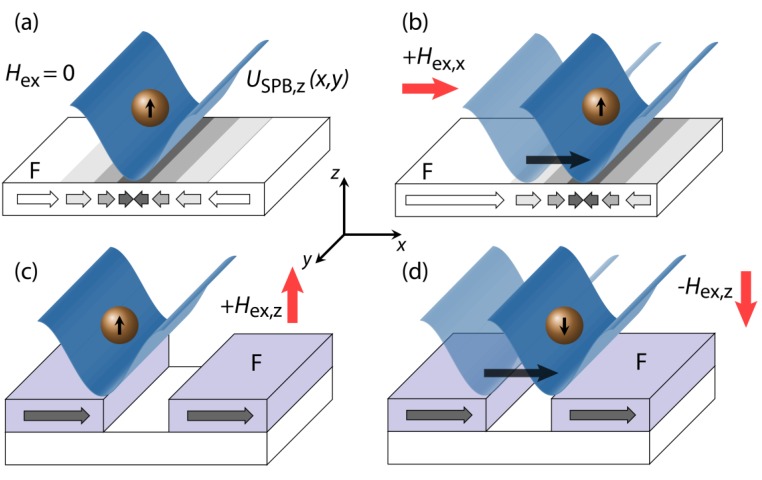
Sketch of the different concepts described in literature for the directed transport of SPBs above magnetically patterned substrates via dynamic transformation of the SPB’s magnetic potential energy landscape $U_{SPB,z}(x,z)$ at a distance z to the substrate surface: (**a**,**b**) SPBs initially located above the domain wall of a ferromagnetic (F) layer (arrows indicate the x-component of the substrate’s magnetization) are transported due to the magnetization reversal of the substrate via domain wall movement in the presence of an external magnetic field $H_{ex,x}$; (**c**,**d**) The superposition of the MFL above topographic micro magnets with a time-dependent external magnetic field causes the dynamic transformation of the SPB’s magnetic potential energy landscape and hence, a directed movement of the SPB in the presence of $H_{ex,z}$ without modification of the substrate’s remanent magnetic state.

Fast transport of SPBs exposed to dynamically varying MFLs has been achieved by domain wall displacement in zig-zag ferromagnetic wire structures [28,29,36,37], or by hopping between topographically elevated micro magnets (see Figure 1) [31,38,39,40,41,42,43,44,45]. Further concepts demonstrated that SPBs can be transported by moving domain walls [46] driven, e.g., in magnetic garnet films by three-dimensional oscillating external magnetic gradient fields [30,47,48], or by introducing asymmetric magnetic potentials, commonly referred to as a magnetic ratchet (Figure 1) [25,48,49]. Although the typical magnetic field strengths emerging from such domains or micro magnets are rather small, the corresponding magnetic field gradients are comparably strong over small distances [50]. Within this distance, therefore, high SPB velocities (in the range of 100 µm/s) are obtained [41,47]. In these concepts, the varying external magnetic field pulse sequence is then used to spatially shift these local magnetic gradient fields across the substrate surface [31,41,47]. Hence, a directed transport of SPBs is induced by the dynamic transformation of the SPBs’ magnetic potential energy landscape [27,31,41].

The technology platform described in this report enables to create MFLs through defined magnetic domain patterns in continuous and essentially flat Exchange-Bias (EB) layer systems. The magnetic domain patterns are tailored by low-energy light-ion bombardment induced magnetic patterning (IBMP) and can be fabricated over whole wafers [51,52,53,54]. Superparamagnetic beads are moved above this material system via two different mechanisms: either by the domain wall movement assisted transport (DOWMAT) at somewhat higher external magnetic field strengths (causing magnetization reversal of the layer system), or by the time-dependent alteration of the effective magnetic field landscape (MFL) above the EB system by comparably weak external magnetic field pulses (without modification of the remanent magnetic state of the substrate) [26,27]. For the use in LOC or µTAS devices, the alteration of the magnetic field landscape by external fields is promising as the necessary fields are small enough to be created by small air coils on-chip. The technology to fabricate the substrates for the SPB transport, the quantitative determination of the stray fields created by the magnetically patterned substrates and the transport of superparamagnetic beads by the alteration of the magnetic field landscape through external magnetic field pulses will be described in the present report, together with some characteristic aspects of the transport mechanism in view of applications.

## 2. Tailored Domains in Exchange-Bias Layer Systems by Low-Energy Light-Ion Bombardment Induced Magnetic Patterning (IBMP)

**Figure 2:**
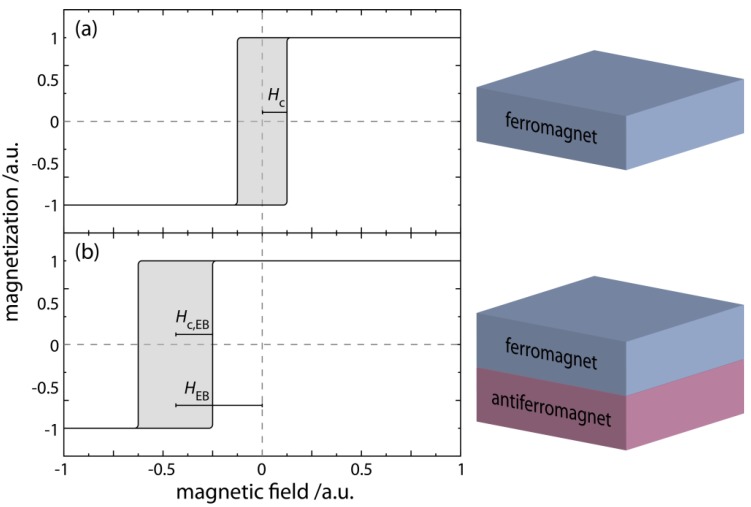
Sketch of the main characteristics of an EB thin film system as compared to an idealized pure ferromagnetic layer (**a**) as seen from the respective hysteresis loops when the measuring field is aligned collinearly to the direction of the easy axis of the ferromagnetic layer (**a**) or the direction of the unidirectional anisotropy of the EB layer system (**b**). The magnetization reversal of the ferromagnetic layer occurs around the coercive field ${\vec{H}}_{c}$, at which the net magnetization of the layer is zero. The hysteresis loop in this case is essentially square shaped. The exchange coupling between the ferromagnet (F) and the antiferromagnet (AF) results in a unidirectional anisotropy, shifting the symmetry point of the hysteresis loop to a finite field along the magnetic field axis and is characterized by the EB field ${\vec{H}}_{EB}$ and an increased coercive field ${\vec{H}}_{c,EB}$.

The magnetic layer system of the technology platform is a continuous Exchange-Bias layer system [55,56,57,58]. The main characteristics of EB layer systems are summarized as follows: an EB layer system consists of a ferromagnetic and an antiferromagnetic layer being in contact to each other. Due to interface exchange coupling between the magnetic moments of the two adjacent layers, a unidirectional anisotropy emerges. The corresponding hysteresis loop essentially exhibits two modifications in comparison to a simple ferromagnetic thin film (Figure 2): a symmetry point at a finite magnetic field (characterized by the Exchange-Bias field ${\vec{H}}_{EB}$) and a change in the coercive field ${\vec{H}}_{c,EB}$, which is typically increased. If both the unidirectional anisotropy is strong enough (${\vec{H}}_{EB}>{\vec{H}}_{c,EB}$) and the corresponding hysteresis curve is almost square shaped, the remanent magnetic state of the ferromagnetic layer is precisely defined.

The EB-effect can be initialized by field growth [59,60], field cooling [56], thermally induced ordering [61], and low-energy light-ion bombardment [62]. There are several models to theoretically describe the EB and the correlated phenomena [55,56,57,58,59,63]; however, a common description which is valid for all different types of material systems is still not available due to the manifold of physical boundary conditions. A presentation of the different models will be out of the scope of this report.

The EB-field (direction and magnitude) and coercive fields of EB layer systems may be altered upon low-energy light-ion bombardment in an applied in-plane external magnetic field ${\vec{H}}_{IB}$ (IBMP). Depending on the ion dose and the properties of the EB system to be investigated, the magnitude of ${\vec{H}}_{EB}$ can be either increased or decreased provided that ${\vec{H}}_{IB}$ is aligned along the initial unidirectional anisotropy direction [64,65].

**Figure 3:**
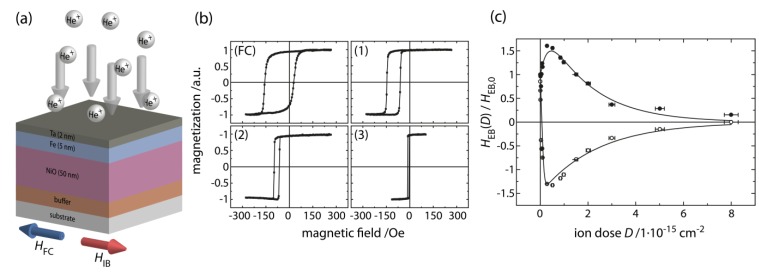
Effects of low-energy (10–30 keV) light-ion bombardment on the EB and coercive fields of an EB layer system. (**a**) Typical EB layer system, where the EB bilayer system (NiO–antiferromagnet; Fe–ferromagnet) is embedded between a buffer and capping layer (Ta). He^+^-ion bombardment is indicated by gray arrows, blue and red arrows indicate the directions of the fields applied during EB initialization by field cooling (${\vec{H}}_{FC}$) and of the field applied during ion bombardment (${\vec{H}}_{IB}$), respectively; (**b**) Measured hysteresis loop of the EB layer system after field cooling (FC). (**1**)–(**3**) Measured loops of the same layer system after bombardment by 3 × 10^14^ (**1**), 1 × 10^15^ (**2**), and 8 × 10^15^ (**3**) 10 keV He^+^-ions/cm^2^. At an ion dose of around 1 × 10^16^ ions/cm^2^ the loop returns to a loop characteristic for the pure ferromagnetic layer; (**c**) Typical dependence of the ratio between ${\vec{H}}_{EB}(D)$, the EB field after bombardment, and ${\vec{H}}_{EB,0}$ as a function of the applied ion dose *D*, where ${\vec{H}}_{EB,0}$ is the EB field after layer deposition and field cooling. Filled circles indicate values when ${\vec{H}}_{IB}$ is parallel to ${\vec{H}}_{FC}$, open circles when ${\vec{H}}_{IB}$ is antiparallel to ${\vec{H}}_{FC}$.

Using ion doses in the range of 1 × 10^16^ ions/cm^2^, the EB-effect is typically destroyed, which is mainly due to both interface intermixing and an overall reduction of the magnetocrystalline anisotropy energy of the antiferromagnetic grains [64,66]. The corresponding hysteresis loop is similar to the one of a pure ferromagnetic system (Figure 3b), although ion implantation may lead to a decreased exchange stiffness of the ferromagnetic layer. For an ion dose ranging between 10^14^ and 10^15^ ions/cm^2^ the EB field is usually enhanced (Figure 3b,c), resulting in typical dose dependencies as displayed in Figure 3c. The underlying physics has been worked out in a series of papers and will not be described here [51,53,62,65,67].

In the case of ${\vec{H}}_{IB}$ being applied antiparallel to ${\vec{H}}_{FC}$, the direction of ${\vec{H}}_{EB}(D)$ can be reversed by ion bombardment and its magnitude is modified in a characteristic way (Figure 3) [51,68,69,70,71,72]. More generally, the direction of the induced unidirectional anisotropy may be set by the direction of the bombardment field ${\vec{H}}_{IB}$ to an arbitrary angle with respect to the unidirectional anisotropy direction of the initial layer system [51,68]. Finally, it has been shown that this technique may be applied to a wide variety of EB layer systems and moreover, different types of light-ions may be used for the defined modification of the unidirectional anisotropy [46,66,70,71,73,74,75,76].

**Figure 4:**
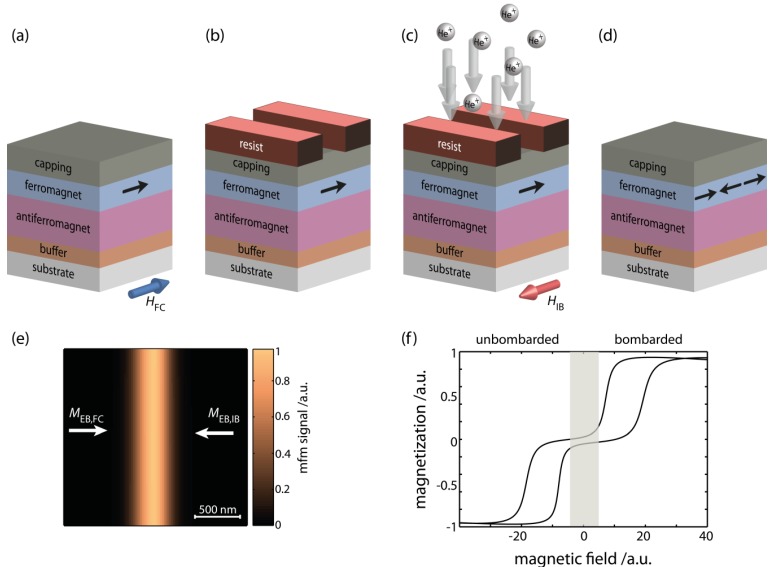
Ion bombardment induced magnetic patterning (IBMP) of EB layer systems by low-energy light-ions to fabricate parallel stripe magnetic domains with antiparallel remanent magnetizations (${\vec{M}}_{EB,FC}$ and ${\vec{M}}_{EB,IB}$) along the short stripe axis. (**a**) EB layer system. The remanent domain’s magnetization due to the unidirectional interface exchange anisotropy is indicated by the black arrow parallel to the direction of the external magnetic field ${\vec{H}}_{FC}$ applied during field cooling; (**b**) Mask for the patterning. The thickness of the mask (resist) prevents the ions to penetrate through it into the magnetic layer system; (**c**) 10 keV He^+^-ion bombardment in an external magnetic field ${\vec{H}}_{IB}$ antiparallel to ${\vec{H}}_{FC}$; (**d**) Magnetically stripe patterned EB layer system after mask removal; (**e**) Generic theoretically predicted magnetic force microscope (MFM) image of the magnetically patterned EB layer system shown for a transition where the remanent domains’ magnetizations meet head-on (adapted with permission from Holzinger *et al.*) [68]; (**f**) Hysteresis loop of the magnetically patterned layer system of (**d**), recorded by vibrating sample magnetometry with the sensitivity direction along the axes of the two antiparallel unidirectional anisotropies.

The combination of the low-energy light-ion bombardment induced modification of the unidirectional anisotropy with lithography techniques or by the use of ion beam writing enables the tailoring of magnetic domains with arbitrarily set in-plane magnetization in remanence [51,68,71,76,77]. In particular, the magnetization direction in the tailored domains can be set independently of the geometry of the domains, which is defined by the geometry of the mask, and the pattern can be fabricated over large surface areas [68]. The whole process has been called ion bombardment induced magnetic patterning (IBMP). Figure 4 shows the major technology steps to create an artificial magnetic domain pattern using a lithography mask. In an EB layer system the unidirectional anisotropy will be initialized via a field cooling procedure (a) and a lithography mask is created on top of the surface of this layer system (b). The thickness of the mask prevents the ions to penetrate into the magnetic layer system. After bombardment by 10 keV He^+^-ions in an external magnetic field ${\vec{H}}_{IB}$ (c) and removal of the resist mask, the magnetic domains are created (d). The surface of the patterned layer system is essentially flat with an average rms roughness of around 1 nm [68,78]. Magnetic patterns may be imaged by magnetic force microscopy (e) or Kerr microscopy and their magnetization reversal may be characterized by Kerr or vibrating sample magnetometry (f) or similar methods [68,78,79,80,81].

**Figure 5:**
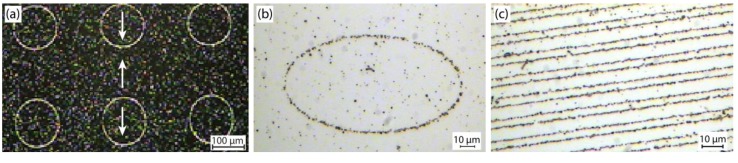
Domain wall decoration of IBMP patterned domains in EB layer systems by SPBs. (**a**) Circular domains with magnetizations pointing upwards and downwards in the rest of the layer system (as indicated by the arrows); (**b**) Magnetization configuration same as in (**a**), but here for an ellipsoidal domain; (**c**) Parallel stripe domains with magnetizations antiparallel to each other and perpendicular to the long stripe axis.

In EB thin films, Néel walls will form between adjacent magnetic domains where inhomogeneous magnetic stray fields emerge due to the spatial divergence of the samples’ magnetization distribution [82]. These stray fields usually attract SPBs in liquids which may serve to visualize the micromagnetic domain structure referring to the well-known Bitter technique [83]. Some examples for domain wall decoration by SPBs [84,85,86] to visualize different geometries of the IBMP fabricated domains are shown in Figure 5.

**Figure 6:**
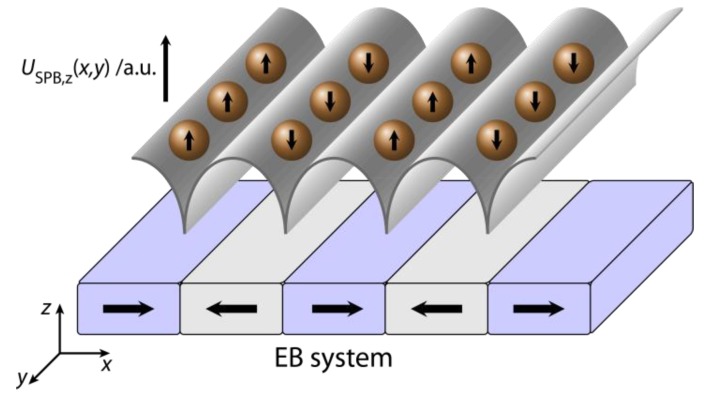
Sketch of the parallel stripe magnetic domain pattern with the corresponding potential energy landscape $U_{SPB,z}(x,y)$ at a distance Δz to the substrate surface. It is obvious that the induced magnetic moment of each SPB above one domain wall is oriented in the same direction causing a mutual repulsion of the SPBs within the stray fields above one domain wall. The distance between two adjacent domain walls is large enough as to keep the mutual magnetic dipolar attraction between SPBs above adjacent walls negligible (adapted with permission from Holzinger *et al.* [27]).

Among the large variety of possible domain geometries and relative magnetization directions IBMP fabricated parallel stripe magnetic domains proved to be useful for the directed transport of SPBs [26,27]. If SPBs are attracted by the inhomogeneous magnetic stray fields above the corresponding domain walls, their magnetic field induced magnetic moments are essentially oriented perpendicular to the substrate’s surface within the magnetic stray field of one domain wall [27]. Due to the parallel alignment of the SPBs’ magnetic moments trapped in the magnetic stray field of the same domain wall, the SPBs magnetostatically repel each other by their magnetic dipolar interaction (Figure 6) [27]. As long as the SPBs are trapped in the stray field of such a domain wall, the mutual magnetostatic repulsion is present, suppressing the agglomeration of SPBs in solution [27].

Using IBMP it is possible to fabricate these domain patterns with long range order regarding the pattern periodicity and orientation over a wafer of several inches [51,68].

## 3. Quantification of Stray Fields above Patterned Exchange-Bias Layer Systems

**Figure 7:**
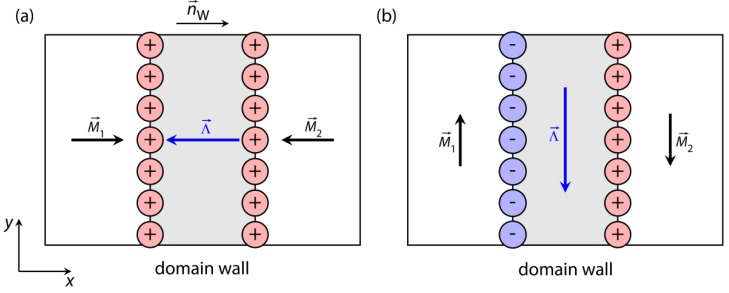
Magnetic charge distribution around Néel walls between domains with antiparallel magnetizations: (**a**) The vectorial difference $\vec{\Lambda }$ between the two magnetizations ${\vec{M}}_{1}$ and ${\vec{M}}_{2}$ is parallel to the normal vector ${\vec{n}}_{W}$ of the domain wall and causes monopolar magnetic charges; (**b**) The vectorial difference is perpendicular to the normal vector of the domain wall and creates dipolar magnetic charges (adapted with permission from Holzinger *et al.* [68]).

The possibility to set the unidirectional anisotropies (and therefore the remanent orientation of the magnetization) in the fabricated magnetic domains independent of the domain geometry is an important feature to optimize MFLs for SPB transport [68]. For fast transport, the gradients within the MFLs must be as large as possible at relevant SPB distances to the substrate surface. For adjacent domains with in-plane magnetizations two extreme cases may be distinguished: (1) magnetizations being antiparallel to each other and their vectorial difference parallel to the domain wall normal vector (Figure 7a); and (2) antiparallel magnetizations with their vectorial difference being perpendicular to the domain wall’s normal vector (Figure 7b) [68].

Case (2) is typical for domain walls without any magnetic net charges [68,82,83]. Looking into this system a bit more closely, dipolar magnetic charges will be created, separated by a distance corresponding to the width of the Néel wall core [83]. The magnetic stray fields of these charges will undergo flux closure over these small distances, and therefore, the corresponding stray fields do not reach out further away from the surface of the layer system. In case (1) monopolar charges will be created, which will be only able to undergo flux closure with the stray fields of the neighboring domain walls (in the case of the parallel stripe magnetic domain pattern) [68,83,87].

As the lateral distance over which the magnetic field varies is given by the domain width, a maximum in the magnetic field gradient is achieved when the magnitude of the magnetic field strength above neighboring domain walls is maximized, assuming that the spatial extent of the domain wall is small in comparison to the distance between two neighboring domain walls. For in-plane magnetized domains in thin films usually Néel walls form between adjacent magnetic domains [83]. The emitted magnetic stray field is proportional to the magnetic net charges σ within the domain wall structure, which is calculated as:

(2) $$\sigma ={\vec{n}}_{w}\cdot \left({\vec{M}}_{1}-{\vec{M}}_{2}\right)$$

where ${\vec{n}}_{w}$ is the unit vector normal to the domain wall plane with its direction defined by going from the first domain with magnetization ${\vec{M}}_{1}$ to the second domain with magnetization ${\vec{M}}_{2}$. $\left({\vec{M}}_{1}-{\vec{M}}_{2}\right)$ is the vectorial difference between the adjacent domains’ magnetizations ${\vec{M}}_{1}$ and ${\vec{M}}_{2}$, respectively [83,87]. Clearly, there is a maximum amount of magnetic net charges, and therefore, a maximum of the corresponding magnetic stray field when the two magnetizations in adjacent domains are oriented antiparallel to each other and their vectorial difference is collinear to the domain wall normal vector. This prerequisite for efficient magnetic particle transport has been investigated in a series of experiments [68]. As a consequence, the optimum domain configuration is achieved by parallel stripe magnetic domains with antiparallel magnetizations in adjacent domains perpendicular to the long stripe axis (Figure 6). Such particular domain pattern can be fabricated by IBMP over a broad surface area, *i.e.*, over the area of a whole wafer. Figure 6 shows the typical domain configuration used for the present particle transport concept and the corresponding potential energy landscape of the SPBs in remanence [27].

For a well-defined movement of SPBs in MFLs with overlaid dynamically varying external magnetic fields, both the magnitude and gradient of the MFLs must be quantitatively known as a function of position above the substrate in the volume where the SPB transport occurs [27,44,50]. As the SPBs are attracted to the substrate’s surface by the inhomogeneous magnetic fields of the MFLs, their distance to the surface is primarily given by the balance of forces between the electrostatic and magnetic force acting on the SPBs when approaching the substrate surface. Therefore, if SPBs of, e.g., 2 µm diameter are used, quantitative knowledge of the MFL within a volume with a height of about 2.5 µm above the surface is necessary. A quantitative magnetic field determination as a function of the three space coordinates in such close proximity to the surface is not at all an easy task. Not only approaching the surface for the measurements at such small distances is a challenge, but also the size of the probe is important when interpreting the measurements. As the probes do determine the magnetic fields as functions of position in a certain height range above the substrate surface, the determination of high gradients within the MFLs implies that the probe has to be as point like as possible in the direction of the magnetic field gradient. For finite-size probes along one or several coordinates, averaging over this lateral (or height) coordinate must be taken into account.

**Figure 8:**
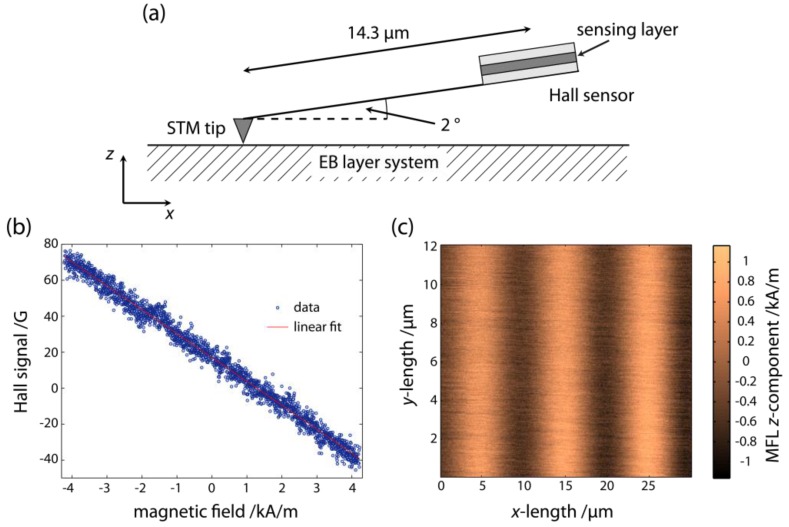
(**a**) Set-up of the scanning Hall probe measurements for the quantitative determination of the MFLs in the volume above the substrate surface relevant for the SPB transport; (**b**) Typical calibration of the Hall probe signal to magnetic field strengths; (**c**) z-component of the magnetic stray field as a function of lateral position at a distance of 750 nm above the substrate surface for a Cu^5 nm^/Ir_17_Mn_83_^30 nm^/Co_70_Fe_30_^12 nm^/Ta^10 nm^ layer system with engineered 5 µm wide parallel stripe magnetic domains. Note that the sensor output averages over the lateral extension of the probe, in this case over 1 µm × 1 µm. The displayed fields are corrected for the 2° tilt of the probe.

As a first approach, we applied scanning Hall microscopy [88,89] with the probe at distances between 0.75 µm and 2.65 µm [50]. The principle of this measurement is shown in Figure 8a. The sensitive probe volume is sheet like with a thickness of 2 nm (two dimensional electron gas within the semiconductor heterostructure of the probe) [90,91] with a spatial extent of 1 µm in the x- and y-direction [50]. Due to the design of the semiconductor heterostructure, the sensing volume is located at a depth of 150 nm from the lower side of the Hall probe [50]. With this measurement geometry, the z-component of the MFL can be measured as a function of the three space coordinates.

The probe is tilted by an angle of 2° with respect to the substrate surface and a scanning tunneling microscopy (STM) tip is attached to it at a distance of 14.3 µm between STM tip and the center of the Hall probe [50]. Measurements are carried out by approaching the surface with the STM tip until contact and then retracting the whole sensor by 100 nm from the substrate surface to avoid a crash of both the STM tip and the Hall probe during the measurement. The minimum Hall probe retraction of 100 nm together with the tilt of the sensor and the location of the sensitive layer in the Hall probe at a depth of 150 nm lead to a minimum achievable measurement plane height above the substrate surface of about 750 nm (Figure 8a) [50]. By retracting the sensor to larger distances from the substrate surface and scanning at each distance along the two lateral coordinates, the Hall signal induced by the MFL is measured in the respective planes parallel to the substrate surface.

The calibration of the Hall probe signal in an applied homogeneous external magnetic field was performed prior to the experiments by using a Helmholtz coil set-up (Figure 8b). For a quantitative determination of the measured z-component of the MFL the Hall probe measurement result must be corrected for two effects: (1) the tilt of the probe by 2° and (2) the averaging over the lateral extension of the probe including the electronic transport regime at which the Hall probe operates, *i.e.*, either ballistic or diffusive [91,92,93]. The first correction is straightforward by dividing by cos(2°) and is therefore almost negligible. Figure 8c shows the Hall probe signal, already converted to magnetic field strength present in the plane at a distance of 750 nm from the substrate surface for parallel stripe (hh) and (tt) magnetized domains of 5 µm widths in an Ir_17_Mn_83_/Co_70_Fe_30_ EB layer system with this correction.

The second correction, however, needs an in-depth discussion. As this type of sensor cannot be made smaller in the used apparatus, a quantitative model for the MFL has been developed, resembling a measurement with an idealized point-like probe. The model MFL is then averaged over the lateral extension of the Hall probe as a function of the lateral coordinates and, subsequently, it is compared to the measurement results. The free parameters of the model are fitted to best agreement between averaged model calculations and measurements. The MFL determined in this way is then regarded as the quantitatively determined MFL [50].

The numerical model used for these calculations will now be described briefly. For the calculation of the MFL above a domain wall between in-plane magnetized domains we start with the magnetic volume charges $\rho_{V}$ due to the spatial divergence of the sample’s magnetization distribution $\vec{M}\left(\vec{r}\right)$ within the volume of the domain wall [83]:

(3) $$\rho_{V}=div\left(\vec{M}\left(\vec{r}\right)\right)$$

These volume charges are the sources of the magnetic stray field above the domain wall. The volume charges may be equivalently described by magnetic interface charges $\rho_{I}$ along the wall (interface) between the two magnetic domains possessing two different magnetization directions [83]. The interface charges created by one of the two adjacent domains are described by:

(4) $$\rho_{I}={\hat{n}}_{W}\cdot {\vec{M}}_{s}$$

where ${\hat{n}}_{W}$ is the unit normal vector of the domain wall plane, where the direction of ${\hat{n}}_{W}$ is again defined by going from the first domain with magnetization ${\vec{M}}_{1}$ to the second domain with magnetization ${\vec{M}}_{2}$, and $M_{s}$ is the saturation magnetization of the ferromagnetic layer. Using either Equations (3) or (4), the magnetic scalar potential $\Phi \left(\vec{r}\right)$ can be calculated as:

(5) $$\Phi \left(\vec{r}\right)=\frac{1}{4\pi }\cdot \int \frac{\rho_{V}\left(\vec{r}\prime \right)}{\left|\vec{r}-\vec{r}\prime \right|}dV\prime =\frac{1}{4\pi }\cdot \int \frac{\rho_{I}\left(\vec{r}\prime \right)}{\left|\vec{r}-\vec{r}\prime \right|}dI\prime$$

where V′ and I′ represent the volume and the surface area of the domain wall plane, respectively [83].

The volume integral of Equation (5) can be solved analytically for a constant volume charge density. Therefore, the magnetic charges around the domain wall have been modelled by a sum of small volume elements, in which the magnetic charge density is assumed to be constant.

Furthermore, as the thickness of the ferromagnetic layer is small as compared to the domain wall width, we neglect the integration along the z-axis. The thickness of the ferromagnetic layer is taken into account by multiplying the volume charge density with the thickness of the ferromagnetic film $t_{F}$, which corresponds to a projection of the magnetic charges onto an infinitesimally thin ferromagnetic layer. The resulting area charge density $\rho_{A}\left(x,y\right)$ is therefore:

(6) $$\rho_{A}(x,y)=t_{F}\cdot \rho_{V,z}(x,y)$$

For parallel stripe magnetic domains there is no change in the magnetic charge density as a function of the y-position. The contribution of a line of length L along the y-axis with constant charge density to the magnetic scalar potential is taken into account for by integration over the length L along the y-axis:

(7) $$\rho_{L}\left(x\right)=\int_{-L/2}^{L/2}\rho_{A}\left(x,y\prime \right)dy\prime$$

**Figure 9:**
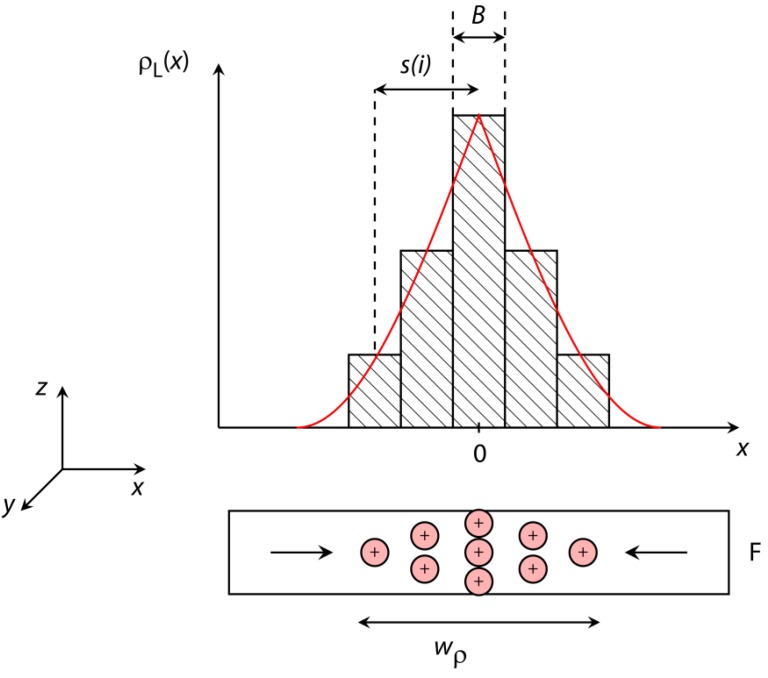
Sketch of the theoretical model developed to quantitatively describe the MFL as a result of the spatial divergence of the samples’ magnetization distribution in terms of the magnetic volume charges inside the domain walls between adjacent domains. The actual magnetic charge distribution $\rho_{L}(x)$ (red line) is modelled by a sum of small volume elements of widths B, where the total width of the magnetic charge distribution within the ferromagnetic layer (F) is described by $w_{\rho }$. s(i) represents the shift of the volume element i with respect to the origin of the coordinate system.

A sketch of this procedure is shown in Figure 9. Since the charge density only varies as a function of the x-coordinate (perpendicular to the domain wall plane), the x-coordinate is now subdivided into $N_{B}$ line elements of width B with individual charge densities.

The sum representation of the integral, therefore, reduces to the following sum of line elements:

(8) $$\Phi \left(\vec{r}\right)=\frac{1}{4\pi }\sum_{i=1}^{N_{B}}\rho_{A}(x=s(i))\cdot \int_{-B/2+s(i)}^{+B/2+s(i)}\int_{-L/2}^{+L/2}\frac{1}{\left|\vec{r}-\vec{r}\prime \right|}dy\prime dx\prime$$

The total charge across a charged (hh) or (tt) domain wall is distributed according to Equation (8) to contribute to the magnetic scalar potential.

As the total charge $\sigma_{tot}$ of the domain wall is known from the characteristics of the layer system, it can be used to deduce the partial line charge densities as occurring in Equation (8):

(9) $$\sigma_{tot}=2\cdot M_{S}\cdot t_{F}\cdot L=t_{F}\cdot \sum_{i=1}^{N_{B}}\rho_{L}\left(x=s\left(i\right)\right)\cdot B\cdot L$$

The samples’ magnetic field landscape ${\vec{H}}_{MFL}(\vec{r})$ can be subsequently obtained via the spatial gradient of the magnetic scalar potential $\Phi (\vec{r})$ according to [83]

(10) $${\vec{H}}_{MFL}(\vec{r})=-\vec{\nabla }\Phi (\vec{r})$$

As an example, the scanning Hall probe microscopy (SHPM) data observed for the EB layer system Cu^5 nm^/Ir_17_Mn_83_^30 nm^/Co_70_Fe_30_^12 nm^/Ta^10 nm^ magnetically patterned into parallel stripe magnetic domains with a magnetic domain width of 5 µm is used to test the numerical model. To take into account the interaction between the stray fields of neighboring domain walls, the inverted stray field signal of the two neighboring tail-to-tail domain walls was first superimposed on the stray field signal of the head-to-head domain wall. For the simulations, a Gaussian charge distribution with a standard deviation of 300 nm was assumed and the length of the domain wall along the y-axis was equated to the sample size of 15 mm.

**Figure 10:**
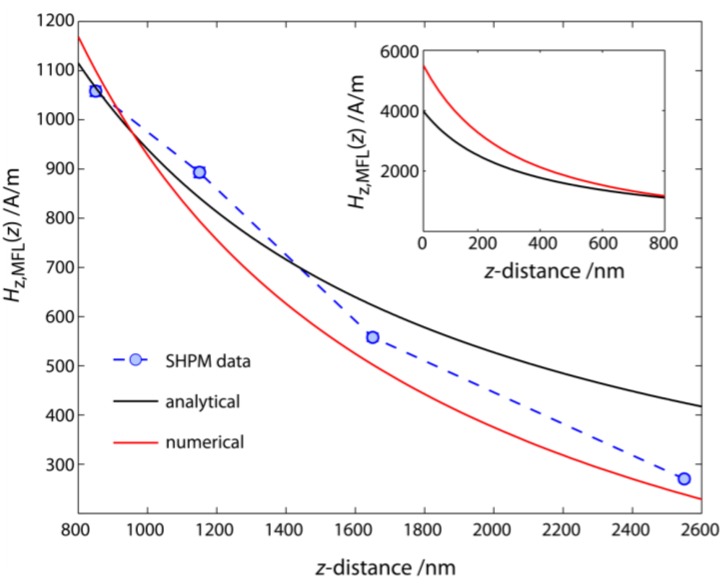
z-component of the samples’ MFL $H_{z,MFL}(z)$ as a function of the z-distance to the substrate surface observed for the EB layer system Cu^5 nm^/Ir_17_Mn_83_^30 nm^/Co_70_Fe_30_^12 nm^/Ta^10 nm^ magnetically patterned into parallel stripe magnetic domains with a magnetic domain width of 5 µm as obtained from the SHPM measurements. The experimental data was fitted by both an analytical model for the description of longitudinal magnetic recording media and by using the numerical approach for the magnetic charge distribution inside the domain wall, where a Gaussian with a standard deviation of 300 nm was assumed for the magnetic charge distribution along the x-axis. The-next-neighbor-interaction between neighboring domain walls was taken into account by superimposing their stray field signal on the stray field signal of the head-to-head domain wall under focus. The dashed line serves as a guide to the eye.

The total number of magnetic charges inside the domain wall, which is proportional to the magnitude of the MFL, was scaled to the SHPM data (linear scale) according to a reduction observed for the saturation magnetization of the Co_70_Fe_30_ film which is mainly due to structural effects (e.g., layer thickness) and temperature [83,94,95,96,97]. The resulting data was compared to the distance dependent progression of the z-component of the samples’ MFL obtained by an analytical model which was initially developed to describe longitudinal magnetic recording media [98,99,100]. The analytical approach is based on the assumption that the spatial change of the x-component of the magnetization within the (hh) and (tt) domain wall can be described by an arctangent function [98,99,100].

As can be seen from Figure 10, the z-component of the measured MFL $H_{z,MFL}(z)$ as determined from the SHPM measurements is described well with the results of both the present numerical approach and the analytical model, respectively. However, the analytical model is restricted to the theoretical description of the MFL above magnetically stripe patterned substrates with (hh) or (tt) magnetized domains whereas the numerical approach is capable to theoretically predict the MFL above almost any type of domain wall.

## 4. Superparamagnetic Bead Transport by External Magnetic Field Triggered Magnetic Stray Field Manipulation

In the present section, a theoretical model will be outlined for the quantitative description of the directed transport of SPB rows above magnetically stripe patterned EB thin film systems. The transport mechanism is based on the dynamic transformation of the SPBs’ magnetic potential energy landscape due to the application of weak external magnetic field pulses, *i.e.*, without affecting the magnetic state of the substrate. As has been shown in Section 3, the spatial distribution of magnetic net charges within the domain walls formed between adjacent domains of magnetically stripe patterned EB layer systems and hence, both the magnitude and the gradient of the corresponding MFL can be specifically tailored by the relative orientation of the neighboring domains’ magnetization vectors ${\vec{M}}_{1}$ and ${\vec{M}}_{2}$ via IBMP as well as by the intrinsic material properties of the ferromagnetic layer, *i.e.*, the saturation magnetization $M_{S}$, the layer thickness $t_{F}$, and the effective magnetic anisotropy parameters [68]. It has been shown that both the magnitude and the spatial gradient of the MFL are maximized for the (hh) and (tt) domain configuration, where the adjacent domains’ magnetization ${\vec{M}}_{1}$ and ${\vec{M}}_{2}$ is parallel and antiparallel to the domain wall normal vector, respectively (see Figure 7). Therefore, this domain configuration will be employed for the developed concept of the directed transport of SPBs.

The potential energy of a superparamagnetic bead $U_{SPB}(x,z)$ exposed to a magnetic field $\vec{H}(x,z)$ possessing a magnetic field induced magnetic moment ${\vec{m}}_{SPB}(x,z)$ at a position x,z above the surface of a magnetically stripe patterned EB layer system - taking into account the translation symmetry of the stripe domain pattern along the y-axis - is given by the inner product:

(11) $$U_{SPB}(x,z)=-\mu_{0}\cdot {\vec{m}}_{SPB}(x,z)\cdot \vec{H}(x,z)$$

where $\mu_{0}$ is the vacuum permeability [34]. SPBs are characterized by a saturation magnetic moment $m_{S,SPB}$ comparable to that of ferromagnetic materials and by their magnetization reversal behavior, which is similar to that of a paramagnetic material, *i.e.*, there will be no net magnetization in remanence (on the timescale of the experiment) [101]. As a result, the SPBs’ magnetic moment can be quantitatively described in a point-particle approximation using the Langevin formalism for paramagnetic materials, which is a function of the SPBs’ saturation magnetic moment $m_{SPB,S}$ and the magnitude of the magnetic field [102]:

(12) $$\left|{\vec{m}}_{SPB}(x,z)\right|=m_{SPB,S}\cdot \left[\coth\left(b\cdot \left|\vec{H}(x,z)\right|\right)-\left(\frac{1}{b\cdot \left|\vec{H}(x,z)\right|}\right)\right]$$

where b is Langevin Parameter, $k_{B}$ is the Boltzmann constant and T is the temperature. Since the SPBs’ magnetic moment is oriented parallel to the direction of the external magnetic field, the x- and z-component of the SPBs’ magnetic moment $m_{SPB,x}(x,z)$ and $m_{SPB,z}(x,z)$ is defined by the angle $\alpha =\arctan\left(H_{z}(x,z)/H_{x}(x,z)\right)$, which is again given by the x- and z-component of the external magnetic field [27]:

(13) $$m_{SPB,x}(x,z)=\cos\left(\alpha \right)\cdot \left|{\vec{m}}_{SPB}(x,z)\right|$$

(14) $$m_{SPB,z}(x,z)=\sin\left(\alpha \right)\cdot \left|{\vec{m}}_{SPB}(x,z)\right|$$

In a microfluidic device where a magnetically stripe patterned EB layer system possessing a (hh) and (tt) domain configuration is used as a substrate generating the MFL, the SPBs’ magnetic moment is solely influenced by the local strength of the MFL at a position x,z above the substrate surface in the absence of an external magnetic field [27]. Therefore, an accurate knowledge concerning the spatial distribution of the MFL’s magnitude and gradient is crucial in order to quantitatively describe the local magnetic forces acting on the SPBs. As has been shown in Section 3, an analytical model which was initially developed for the description of the MFL above longitudinal magnetic recording media can be used to theoretically predict the distance dependence of the x- and z-component of the MFL ${\vec{H}}_{MFL}(x,z)$ above the magnetically stripe patterned EB layer system [50]. The analytical model is based on the assumption of an arctangent transition for the x-component of the domains’ magnetization $M_{x}(x)$ inside the domain walls, *i.e.*, perpendicular to the long stripe axis [50,98,99,100]. As a second boundary condition, the adjacent domains’ magnetization is given by the remanent domain magnetization $M_{r}$ (here in cgs-units) which is related to the saturation magnetization $M_{S}$ of the ferromagnetic layer in a way that is primarily a uniform domain magnetization parallel to the x-axis and a decrease of $M_{S}$ as a function of temperature, layer thickness and a change in both the ferromagnetic layer’s exchange stiffness and anisotropy parameters. Under the further assumption that the remanent domain magnetization is equal in adjacent domains, $M_{x}(x)$ can be written as:

(15) $$M_{x}(x)=\pm \frac{2\cdot M_{r}}{\pi }\cdot \arctan\left(\frac{x}{a}\right)$$

where a is the domain transition parameter, which is related to the domain wall transition length $l_{w}$ by $l_{w}=\pi \cdot a$ [27,50,98,99,100]. The magnetization transition for a series of parallel stripe magnetic domains is described by a sum representation by taking into account the individual displacement of the domain transition given by the parallel stripe’s domain width and whether the transition is of (hh) or (tt) type [27]. The MFL’s x- and z-component above an individual domain transition, $H_{MFL,x}(x,z)$ and $H_{MFL,z}(x,z)$ respectively, can be analytically described by:

(16) $$H_{MFL,x}(x,z)=4\cdot M_{r}\cdot \left[\arctan\left(\frac{x\cdot \left(t_{F}+z\right)}{x^{2}+a^{2}+a\cdot \left(t_{F}+z\right)}\right)-\arctan\left(\frac{x\cdot z}{x^{2}+a^{2}+a\cdot z}\right)\right]$$

(17) $$H_{MFL,z}(x,z)=2\cdot M_{r}\cdot \ln\left(\frac{x^{2}+{\left(t_{F}+z+a\right)}^{2}}{x^{2}+{\left(z+a\right)}^{2}}\right)$$

where $t_{F}$ is again the thickness of the ferromagnetic layer [27,50,99,100]. The experimental characterization of the MFL’s z-component as a function of the z-distance above the center of a domain wall between adjacent parallel stripe magnetic domains via scanning Hall probe microscopy can be used to determine both the sample’s remanent domain magnetization and the domain transition parameter when Equation (17) is used for nonlinear regression of the experimental data by taking into account the thickness of the ferromagnetic layer [50].

The quantitative knowledge of the MFL above the magnetically stripe patterned EB layer system can be subsequently used to calculate both the SPB’s magnetic moment and the magnetic potential energy landscape as a function of the effective magnetic field ${\vec{H}}_{eff}(x,z,t)$, which is a superposition of the sample’s MFL and the time-dependent homogeneous external magnetic field ${\vec{H}}_{ex}(x,z,t)$ [27]. The magnetic force ${\vec{F}}_{M}(x,z,t)$ acting on a superparamagnetic bead at a position x,z above the substrate surface at the time t is given in point-particle approximation by the negative spatial gradient of the SPB’s magnetic potential energy landscape according to [33,34]:

(18) $${\vec{F}}_{M}(x,z,t)=-\vec{\nabla }U_{SPB}(x,z,t)=\mu_{0}\cdot \left({\vec{m}}_{SPB}(x,z,t)\cdot \vec{\nabla }\right)\cdot {\vec{H}}_{eff}(x,z,t)$$

In Equation (18) it is assumed that no change in the EB layer system’s magnetic state is induced under the influence of the external magnetic field sequence applied for the directed transport of SPBs [27]. In a liquid environment, the SPBs are accelerated along the x-axis, *i.e.*, perpendicular to the magnetic parallel stripe domain’s long stripe axis, due to the spatial gradient of the SPBs potential energy landscape in x-direction until the balance of forces between the x-component of both the magnetic force (Equation (18)) and the drag force $F_{R,x}(x,z)$ of the fluid has established, given by [26]:

(19) $$\rho_{SPB}\cdot V_{SPB}\cdot \frac{d\;v_{SPB,x}}{dt}=F_{M,x}(x,z)-F_{R,x}(x,z)$$

In Equation (19) $\rho_{SPB}$ and $V_{SPB}$ are the density and the spherical volume of the SPBs, which is related to the hydrodynamic radius $r_{SPB}$, respectively, and $v_{SPB,x}$ is the x-component of the SPB velocity. For a small Reynolds number laminar flow liquid, the drag force can be expressed by the Stokes law and taking into account the drag coefficient $f_{R}(z,r_{SPB})$, which is a good approximation for common microfluidic devices

(20) $${\vec{F}}_{R}(x,z)=6\cdot \pi \cdot r_{SPB}\cdot \eta_{L}\cdot f_{R}(z,r_{SPB})\cdot {\vec{v}}_{SPB}(x,z)$$

where $\eta_{L}$ is the viscosity of the fluid and ${\vec{v}}_{SPB}(x,z)$ is the steady state velocity of the SPBs [27,103]. Since the drag coefficient is a function of both the distance between the substrate and the SPB surface as well as the hydrodynamic radius of the SPBs, respectively, two different cases may be distinguished: For a SPB moving close to the substrate surface, *i.e.*, z→0, the drag coefficient is maximum with a value of $f_{R}(z\to 0,r_{SPB})=3$ [27]. However, if the SPB is moving far away from the container surface, *i.e.*, within the bulk liquid phase where z→∞, the drag coefficient is $f_{R}(z\to \infty ,r_{SPB})=1$, which describes the transition to the common form of Stokes law [27]. More generally, $f_{R}$ exponentially decays as a function of the z-distance into the bulk of the liquid. For the SPB liquid system used in the first proof-of-principle experiments with $r_{SPB}=$1 µm, the characteristic distance at which $f_{R}$ is half of its maximum value is given for a SPB surface to substrate distance of 70 nm [27]. The characteristic timescale τ over which the acceleration of the SPBs takes place is obtained by solving the equation of motion defined by Equation (19), which leads to the following expression [26]:

(21) $$\tau =\frac{2}{9}\cdot \frac{\rho_{SPB}\cdot r_{SPB}^{2}}{\eta_{L}\cdot f_{R}(z,r_{SPB})}$$

In the present work, distilled water was used as a liquid with a density of $\rho_{SPB}=$1100 kg/m^3^, a viscosity of $\eta_{L}=$1.0093 × 10^−3^ Pa·s at T=293.15 K, and a drag coefficient that was assumed to be $f_{R}=$1.5, *i.e.*, the SPBs are moving in close proximity to the substrate surface [27]. Hence, a value of τ≈200 ns is obtained, *i.e.*, the SPBs achieve almost 99 % of their steady state velocity during the time of 5⋅τ=1 µs. As a result, the SPBs’ steady state velocity can be calculated via the balance of forces between the magnetic and drag force in case the effective magnetic field is temporally constant, *i.e.*, $dH_{eff}(x,z,t)/dt=0$ [27]:

(22) $${\vec{v}}_{SPB}(x,z)=\frac{\mu_{0}\cdot \left({\vec{m}}_{SPB}(x,z)\cdot \vec{\nabla }\right)\cdot {\vec{H}}_{eff}(x,z)}{6\cdot \pi \cdot r_{SPB}\cdot \eta_{L}\cdot f_{R}\left(z,r_{SPB}\right)}$$

According to Equation (22), the SPB steady state velocity is dramatically influenced by both the magnitude and gradient of the effective magnetic field, the viscosity of the surrounding fluid as well as by the material properties (size, magnetic content) and the z-distance of the SPBs to the substrate surface [27]. For the z-distance dependence of the drag coefficient and the EB layer system’s MFL a competitive behavior is observed related to their influence on the magnitude of the SPB steady state velocity. Due to the exponential decay of $f_{R}$ with increasing z-distance, the magnitude of ${\vec{v}}_{SPB}$ increases, whereas both the MFL’s magnitude and gradient decrease while increasing the distance between the SPBs and the substrate, which leads to a decrease of the SPB velocity. Hence, a SPB distance to the substrate surface dependence for the steady state velocity results, which is characteristic for the SPB-surface system with a maximum steady state velocity at a certain distance to the substrate surface [27].

Besides the magnetic forces acting on the SPBs due to inhomogeneous effective magnetic fields above magnetically stripe patterned EB layer systems, the surface forces including both the interaction between the SPBs themselves and the SPBs and the substrate surface, respectively, expressed by the DLVO theory, are most significant when starting from hydrodynamic boundary conditions applied to the microfluidic device [104,105]. The DLVO theory combines the overall attractive van-der-Waals interaction with the electrostatic forces as a consequence of the different zeta potentials ζ of the SPBs and the substrate surface, *i.e.*, the effective surface potential in solution at the shear plane [104]. The zeta potential thereby describes the transition region at which the thermal energy is sufficient to prevent further immobilization of charged ions from solution onto the SPB or substrate surface [104]. Typically, if the zeta potential of both the substrate and the SPBs is around ζ≈ −35 mV, repulsive forces ${\vec{F}}_{el}(z)$ of $\left|{\vec{F}}_{el}(z)\right|\geq$ 1 pN are obtained for a SPB surface to substrate surface distance of z≤ 500 nm, whereas the overall attractive van der Waals force ${\vec{F}}_{vdW}(z)$ is $\left|{\vec{F}}_{vdW}(z)\right|\geq$ 2 pN for a SPB surface to substrate surface distance of z≤ 50 nm in the liquid distilled water [27,105]. Hence, the actual distance between the SPBs and the substrate surface is strongly influenced by the balance of forces between the attractive magnetic force due to the effective magnetic field landscape above the EB substrate surface and the electrostatic repulsion between the surfaces present for a specific substrate-SPB-liquid system. By comparison, the gravity and buoyancy forces, ${\vec{F}}_{G}$ and ${\vec{F}}_{B}$ respectively, as well as inertia forces ${\vec{F}}_{I}$ are almost negligible for the here presented transport concept, since these forces are typically in the range of 50 fN or even less [27].

For the first transport experiments the EB layer system Cu^50 nm^/Ir_17_Mn_83_^10 nm^/Co_70_Fe_30_^6.5 nm^/Ta^10 nm^ was used, which was magnetically patterned via IBMP into parallel stripe magnetic domains with a (hh) and (tt) domain configuration and a regular stripe width of 5 µm. The photoresist stripe pattern used for the IBMP procedure with an average height of 700 nm was preserved to keep the SPBs in a defined height range above the substrate surface where measurements of the substrates’ MFL are quantitatively available via scanning Hall probe microscopy [27]. During the experiments, a trapezoidal external magnetic field sequence was applied along the x- and z-direction with a magnitude of 320 A/m and 1640 A/m, respectively, and a magnetic field alteration rate of $f_{H}=3.2\times 10^{6}$ A/(m·s) during the time periods of increasing and decreasing magnetic field. Since the SPBs pass the 5 µm gap between the neighboring stripes of the photoresist structure during an average time of 125 ms, the distance they are falling along the z-direction due to the imbalance of the gravity and buoyancy force acting on the SPBs was determined to Δz= 25 nm. As this distance is negligible related to the SPB diameter of $d_{SPB}=$ 2 µm, the SPBs will essentially move at a minimum SPB surface to substrate distance of 700 nm above the trenches of the resist structure where the drag coefficient is $f_{R}\approx 1$ [27].

Since the drag coefficient was periodically modulated for these first proof-of-principle experiments, the photo resist stripe structure was replaced in a second series of experiments by a homogeneous resist film with a thickness of 700 nm for a quantitative study of the SPB transport phenomenon. The individual steps during the transport process will be outlined in the following [27].

**Figure 11:**
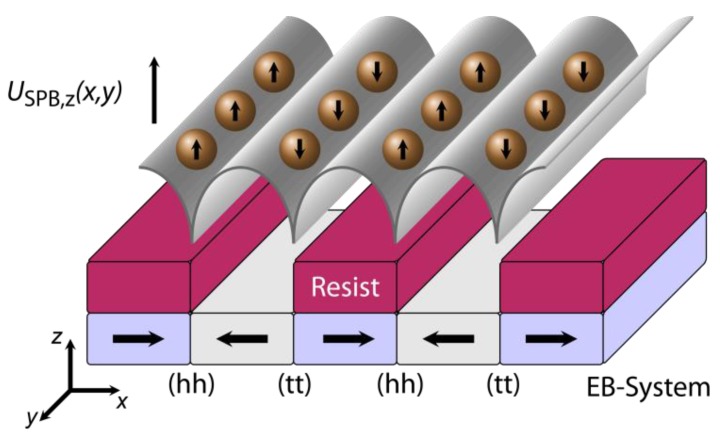
Sketch of the SPBs’ magnetic potential energy landscape $U_{SPB,z}(x,y)$ at a distance z parallel to the surface of the magnetically stripe patterned EB thin film layer system with (hh) and (tt) orientation of the remanent domain magnetization in adjacent magnetic domains perpendicular to the long stripe axis. The SPBs’ magnetic moment is aligned parallel to the local direction of the MFL above the EB substrate. The photoresist structure of the IBMP process was preserved for a feasibility study of the transport concept in order to keep the SPBs at a defined distance above the substrate surface (reprinted with permission from Holzinger *et al.* [27]).

Without an external magnetic field, SPBs from solution first sediment towards the substrate and are simultaneously attracted by the inhomogeneous magnetic stray fields above the (hh) and (tt) domain walls formed between the oppositely magnetized adjacent magnetic domains of the EB layer system (Figure 11).

In this process, the actual distance between the SPBs and the substrate surface is mainly determined by the balance of forces between the attractive magnetic force and the repulsive electrostatic force as a result of the involved surface potentials of the SPBs and the substrate, respectively. The SPBs are accumulated above both the (hh) and (tt) walls, since the corresponding minima of their magnetic potential energy are almost energetically degenerated (Figure 11). Therefore, an initial array arrangement of SPBs is observed with a regular distance of 5 µm between adjacent SPB rows, which is related to the domain width of the parallel magnetic stripe domain pattern of the EB layer system.

Since the SPBs’ magnetic moment is oriented parallel to the MFL above the EB stripe domain pattern, *i.e.*, perpendicular to the substrate surface and parallel for neighboring SPBs along an individual SPB row, magnetostatic repulsion between the SPBs along the SPB rows is observed. The magnetostatic force ${\vec{F}}_{MS}$ is $\left|{\vec{F}}_{MS}\right|\geq$ 10 nN for an inter-particle distance $\vec{r}$ of $\left|\vec{r}\right|\leq$ 1 µm, assuming that the SPBs’ magnetic moment is approximated by the SPBs’ saturation magnetic moment. As a result, SPB agglomeration is strongly avoided along the individual SPB rows [27].

The SPB transport is induced by the dynamic transformation of the SPBs’ magnetic potential energy landscape due to the superposition of the static MFL above the magnetically stripe patterned EB layer system and a designed time dependent external magnetic field pulse sequence. The pulse sequence consists of pulses along the x- and z-axis with a phase shift between these two components of π/2, *i.e.*, a quarter period length. The given field alteration rate of the external magnet in combination with the maximum field amplitude results in rise and fall times of the external magnetic field within a time period of 500 µs. Related to the experimentally observed steady state velocity of the SPBs, the SPBs may move a maximum distance of 20 nm during these periods, which is negligible compared to the distance of 5 µm for one transportation step. Therefore, the transport mechanism can be explained essentially by the SPB motion during the plateau times of the external magnetic field sequence, *i.e.*, where $H_{ex,x}(t)=\pm H_{x,\max}$ and $H_{ex,z}(t)=\pm H_{z,\max}$.

**Figure 12:**
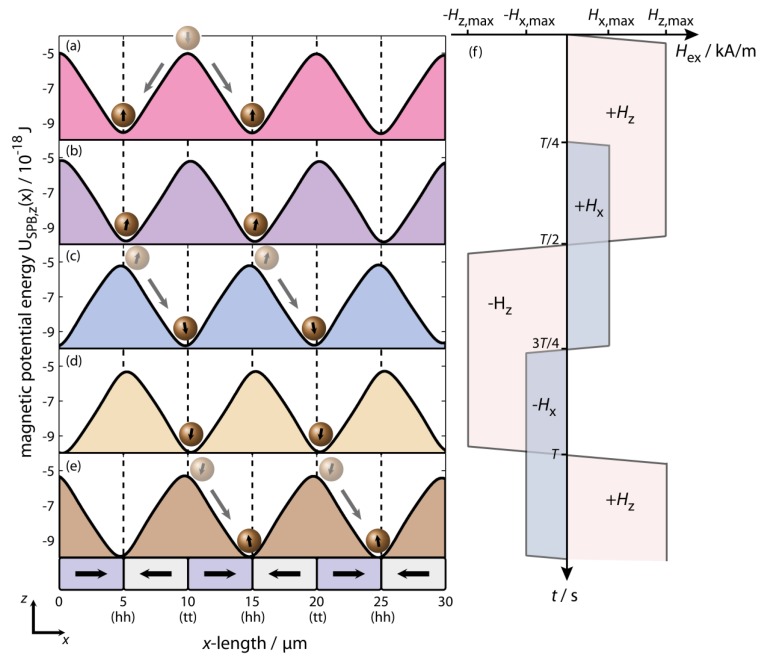
(**a**–**e**) Numerical simulations of the SPBs magnetic potential energy landscape $U_{SPB,z}(x)$ above the magnetically stripe patterned EB layer system perpendicular to the long stripe axis at a distance of 1 µm between the SPB surface and the surface of the EB substrate shown for different configurations of the trapezoidal external magnetic field, *i.e.*, where $H_{x}(t)=\pm H_{x,\max}$ and $H_{z}(t)=\pm H_{z,\max}$ (**f**). The directed transport of SPBs is induced via the dynamic transformation of the SPBs’ magnetic potential energy landscape during a change of sign in $H_{z,\max}$ as indicated by the two generic SPBs; whereas the conversion of $H_{x,\max}$ effects a z-distance dependent change in the potential energy minimas’ x-position along the ±x-direction related to the sign of $H_{x,\max}$. The transport direction is defined by the phase relationship between $H_{x}(t)$ and $H_{z}(t)$ (reprinted with permission from Holzinger *et al.* [27]).

Numerical calculations of the SPBs’ magnetic potential energy landscape were carried out for a SPB center to EB substrate distance of 2 µm for different external magnetic field configurations $H_{x}=\pm H_{x,\max}=\pm$320 A/m and $H_{z}=\pm H_{z,\max}=\pm$1640 A/m, respectively, according to the above described theoretical model (Figure 12). During the initial quarter cycle time period t<T/4 only the external field in z-direction is applied which is superposed with the EB substrate’s MFL.

Since the magnetic stray field signal above the magnetic charge distribution of neighboring (hh) and (tt) domain walls is inverted, the initial degeneration of the SPBs’ magnetic potential energy minima is suspended by the superposition of the EB layer system’s MFL and $H_{z,\max}$, which is caused by the periodic constructive and destructive superposition of these fields. Therefore, SPBs initially located above the (tt) domain walls will statistically move along the ±x-direction into the neighboring potential energy minima above the (hh) domain walls with their steady state velocity which correlates to the spatial gradient of the SPBs’ magnetic potential energy landscape (Figure 12). After the first stochastic transport step, all SPBs are then located above the (hh) domain walls which allows a simultaneous remote control of all SPBs for the following steps. As a further result, the initial distance between neighboring SPB rows is increased to a value of twice the magnetic domain width of the EB stripe domain pattern, *i.e.*, from 5 µm to 10 µm.

For t>T/4, both $H_{x}=\pm H_{x,\max}$ and $H_{z}=\pm H_{z,\max}$ are present and therefore four different configurations during the external magnetic field sequence can be distinguished which will be then periodically repeated: (1) $+H_{x,\max}$, $+H_{z,\max}$; (2) $+H_{x,\max}$, $-H_{z,\max}$; (3) $-H_{x,\max}$, $-H_{z,\max}$; (4) $-H_{x,\max}$,$+H_{z,\max}$ (Figure 12) [27]. The simultaneous superposition of both $\pm H_{x,\max}$ and $\pm H_{z,\max}$ causes a z-distance dependent shift of the potential energy minima (in the present case ±200 nm) with respect to the domain wall center ((hh) or (tt) type) of the magnetically stripe patterned EB layer system, which strongly depends on the magnitude of $\pm H_{x,\max}$ when both $\pm H_{z,\max}$ and the z-distance of the SPBs to the substrate surface are fixed. Furthermore, a slight tilt of the SPBs’ magnetic moment is observed in relation to the surface normal vector. As a result, the effective transport distance between two neighboring domain walls is reduced by a factor of two times the shift.

The directed transport of SPBs proceeds during the plateau times of $\pm H_{z,\max}$ and is induced by the sign change of $H_{z,\max}$ [27].

Thus, the SPBs will move into the nearest potential energy minima with their steady state velocity induced by the spatial gradient of the magnetic potential energy landscape as long as the time the SPBs need to travel between neighboring domain walls is shorter or equal to the plateau time of the external magnetic field sequence, *i.e.*, half the cycle duration [27]. During the plateau times of $\pm H_{z,\max}$, the SPB velocity is dramatically influenced by both the MFL’s and the external magnetic field’s magnitude, the SPBs’ magnetic properties, the viscosity of the surrounding fluid as well as by the z-distance of the SPBs’ to the substrate surface. Since the movement of the potential energy minimum proceeds at an average velocity of 10 mm/s according to the chosen alteration rate of the external magnetic field sequence, which is much faster in comparison to the experimentally observed SPB velocity of 40 µm/s, the SPB velocity is primarily determined by the spatial gradient of the SPBs’ magnetic potential energy landscape after the sign switching of $H_{z,\max}$ [27].

In this concept, the movement direction of the SPBs is defined by the sign of $H_{x,\max}$ when the sign of $H_{z,\max}$ is inversed, since the corresponding minima of the SPBs’ magnetic potential energy are either shifted to smaller or larger x-coordinates with respect to the domain wall center from which the transport step starts. In this concept the SPB steady state velocity $v_{SPB}$ should be independent of the external magnetic fields’ driving frequency $\omega_{ex}$, which has been experimentally proven by estimating $v_{SPB}$ as a function of $\omega_{ex}$ [27].

**Figure 13:**
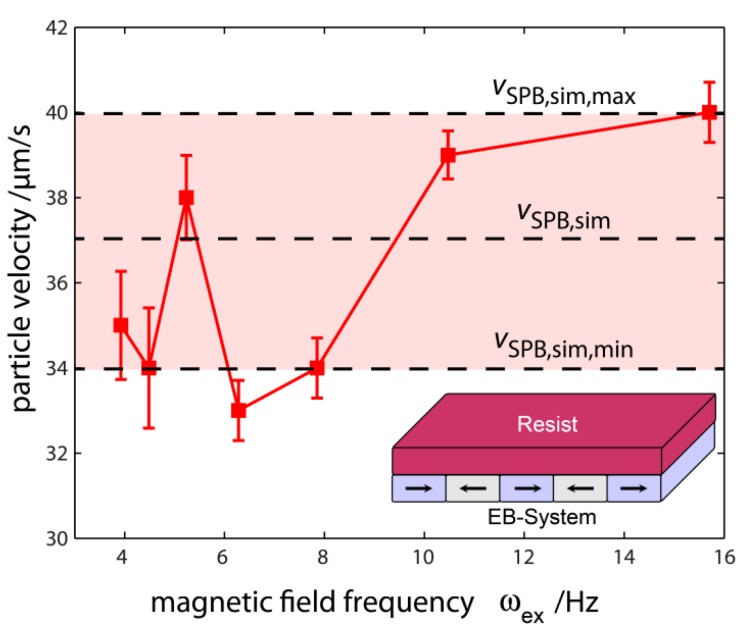
Experimentally determined SPB steady state velocity as a function of the driving frequency $\omega_{ex}$ of the trapezoidal external magnetic field sequence with $H_{x,\max}=320$ A/m and $H_{z,\max}=1640$ A/m for a SPB diameter of 2 µm. In this series of experiments, the magnetically stripe patterned EB layer system was covered by a 700 nm photo resist layer. The theoretically predicted SPB velocity $v_{SPB,sim}$ was obtained at a SPB surface to resist distance of 320 nm. The uncertainty of the numerical simulation, $v_{SPB,sim,\min}$ and $v_{SPB,sim,\max}$, respectively, was estimated by changing the Co_70_Fe_30_ layer thickness in the range of $t_{F}=6.5\pm 0.5$ nm in the calculation of the substrate’s MFL by using the analytical approach. Note that above the critical frequency of $\omega_{ex}\approx 16$ Hz no directed transport of SPBs was observed (reprinted with permission from Holzinger *et al.* [27]).

From Figure 13 it is obvious that the average SPB velocity of 36 ± 4 µm/s is almost independent of the driving frequency $\omega_{ex}$ [27]. For ω>16 Hz no directed transport of SPBs was observed, since the time the SPBs need to travel between neighboring domain walls with their average steady state velocity of 36 µm/s is about 140 ms.

This corresponds to approximately half the cycle duration at 16 Hz when fabrication process induced local fluctuations of both the magnitude and gradient of the EB substrate’s MFL are taken into account [27]. Therefore, if $\omega_{ex}$ is increased above this critical frequency $\omega_{ex,crit}$, the potential energy minimum starts to move before the SPB has reached its final position in the proximity of the neighboring domain wall with the result that the phase correlation between the movement of the SPBs and the movement of their potential energy minima breaks down [27].

The experimentally observed steady state SPB velocity was compared to numerical simulations by using the above described theoretical model [27].

**Figure 14:**
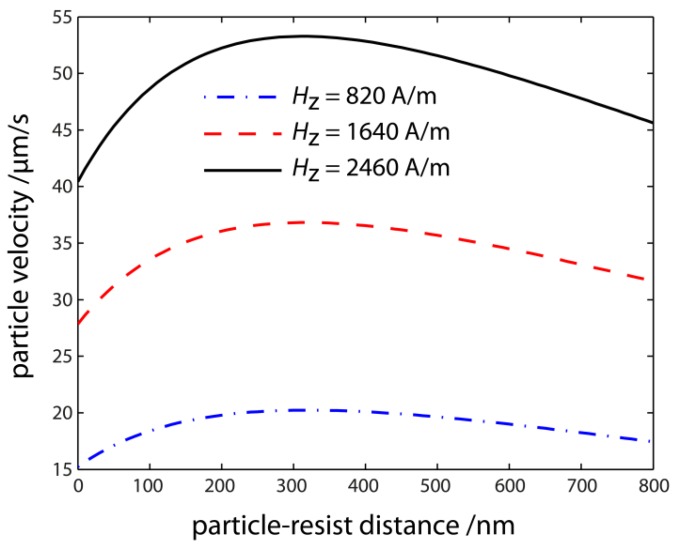
Numerical simulations of the SPB steady state velocity as a function of the z-distance between the SPB and resist surface with a layer thickness of 700 nm shown for different magnitudes of the $H_{z}$-component of the external magnetic field sequence, where the $H_{x}$-component was fixed to $H_{x}=320$ A/m (reprinted with permission from Holzinger *et al.* [27]).

The uncertainty of the theoretically predicted SPB velocity was estimated by taking into account local fluctuations of the EB layer system’s MFL by changing the Co_70_Fe_30_ layer thickness in the range of $t_{F}=6.5\pm 0.5$ nm, expressed in terms of $v_{SPB,sim,\min}$ and $v_{SPB,sim,\max}$. In Figure 14 the calculated SPB velocity is shown as a function of the z -distance between the SPB and resist surface for a fixed x-component of $H_{x}=320$ A/m and several values of $H_{z}$. As can be seen, the SPB velocity first increases with increasing SPB resist distance, which is explained by the more rapid decrease of the drag coefficient $f_{R}$ in comparison to the z-component of the EB substrate’s MFL [27].

The theoretically predicted SPB velocity is maximum at a distance of 320 nm for the experimental parameters of the external magnetic field and subsequently exponentially decreases by further increasing the SPB resist distance. As the SPBs are obviously not sticking to the resist surface during the experiments, it is assumed that the SPBs will move at an average z-distance of 320 nm between the SPB and resist surface with their maximum theoretically predicted velocity. Overall, the theoretically predicted SPB velocity of 37 ± 3 µm/s is in quantitative agreement to the experimentally observed data. Furthermore, the numerical simulations predict a further increase (decrease) of the SPB steady state velocity by increasing (decreasing) the magnitude of the z-component of the external magnetic field sequence. Also, the distance at which the theoretically predicted SPB velocity is maximum is almost constant while changing the magnitude of $H_{z}$ which is due to the unaffected gradient of the effective magnetic field if a homogeneous external magnetic field is applied and the SPBs’ magnetic moment is changed within the linear regime [27]. Note that in the above presented calculations, the magnitude of the SPBs’ magnetic potential energy is always three orders of magnitude larger in comparison to thermal energy at room temperature, *i.e.*, the directed motion of SPBs dominates over the stochastic thermal motion [27].

**Figure 15:**
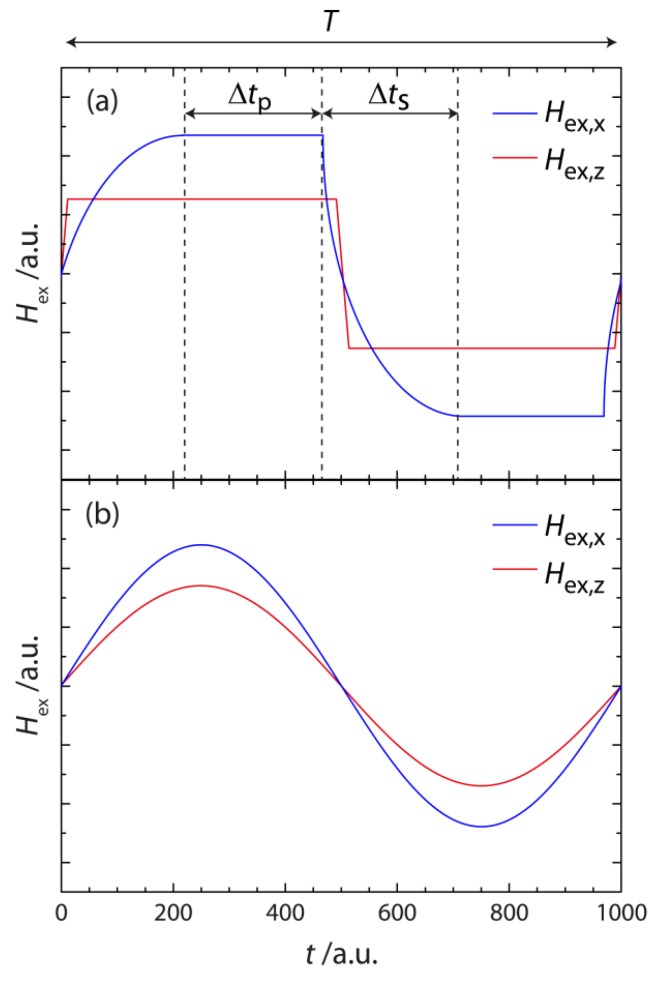
Sketch of the different pulse shapes of the external magnetic field $H_{ex}$ used to investigate their effect on the SPBs’ steady state velocity. In (**a**,**b**) both the x - and z-component $H_{ex,x}$ and $H_{ex,z}$, respectively, are shown for the rectangular (**a**) and sinusoidal (**b**) pulse shape within a cycle duration of T. Here, $\Delta t_{p}$ is the plateau time of the pulse and $\Delta t_{s}$ is the switching time for the magnetic field change between the negative and positive maximum value and vice versa. Note that the increased switching time for $H_{ex,x}$ in (**a**) in comparison to $H_{ex,z}$ is due to the iron core of the coil set-up.

In a further series of experiments, the SPB velocity was investigated as a function of the pulse shape of the external magnetic field sequence applied for the directed transport of SPBs above the magnetically stripe patterned EB substrate. For rectangular field pulses (infinitely fast rise of the magnetic field to its maximum value) of a given maximum magnetic field, the average SPB steady state velocity should not depend on the pulse lengths, as long as the frequency of the external magnetic field does not exceed the critical frequency $\omega_{ex,crit}$ above which the field pulse length is shorter than the time the SPBs need to travel the distance of one domain width with their induced steady state velocity. However, pulses with an infinitely steep rise for the magnetic field strength cannot be realized. Therefore, the dependence of the SPB steady state velocity on the field pulse lengths has been investigated in addition to the experimental results presented in Figure 13 for two different experimentally feasible pulse shapes. In Figure 15 the two different pulse shapes used in the present experiments are shown, *i.e.*, external magnetic field pulses with either rectangular or sinusoidal shape. Since for the following experiments magnetically stripe patterned EB layer systems were used without further resist coating on top of the capping layer, stronger external magnetic field pulses have to be applied according to Figure 10 in order to appropriately manipulate the SPBs’ magnetic potential energy landscape in terms of high achievable SPB velocities in the range of 100 µm/s. As a consequence, a different external magnetic field set-up was used in contrast to the above experimental studies where magnetic fields were solely prepared via air-cored solenoids.

For the periodic rectangular pulses of cycle duration T two different time intervals can be distinguished: During the time $\Delta t_{s}$ the external magnetic field switches from the negative to the positive maximum value and vice versa whereas the plateau time $\Delta t_{p}$ describes the times when the magnetic field is at the maximum value (Figure 15). The rectangular field pulse has exponentially increasing and decreasing pulse edges, which, for longer pulses, resemble more and more a rectangular pulse shape, since the percentage share of the switching time related to the plateau times decreases.

In Figure 15a it is clearly visible that the x-component of the externally applied magnetic field originates from an iron-cored solenoid in the present set-up, whereas the z-component originates from air-cored solenoids as the characteristic time for the magnetic field change is much shorter for the z-component than the one for the x-component. The shortest possible pulse period where the set maximum field strength could be reached was about T≈120 ms, where $\Delta t_{s}\approx 60$ ms and $\Delta t_{p}\approx 0$ ms, defined by the increase time of the x -component of the magnetic field. Shorter pulse lengths did not achieve the set maximum field value with the current set-up and, henceforth, would lead to different magnetic gradient fields for the SPB motion. The maximum field values for the sinusoidal pulses of the x- and z-components of the external magnetic field were chosen to be the same as for the rectangular pulses. Since the magnetic field rise in the sinusoidal pulses with periods larger than T=120 ms is slow, backlash effects are negligible for this pulse form.

These two pulse shapes have been investigated on their effect on the achievable steady state velocity of the SPBs. Table 1 displays the measured SPB steady state velocities within one transport step when the external magnetic fields’ pulse schemes of Figure 15 were applied. For the rectangular pulse scheme, the x- and z-component of the external magnetic field were chosen to be $H_{x}=$ 12 kA/m and $H_{z}=$ 5 kA/m, whereas for the sinusoidal shape $H_{x}$ and $H_{z}$ were set to $H_{x}=$ 24 kA/m and $H_{z}=$ 16 kA/m, respectively. In the experiments, the EB layer system Cu^50 nm^/Ir_17_Mn_83_^10 nm^/Co_70_Fe_30_^7.5 nm^/Ta^10 nm^ was used, which was magnetically patterned via IBMP into parallel stripe magnetic domains with a (hh) and (tt) domain configuration and a regular stripe width of 5 µm. Again, a suspension of SPBs in distilled water with a SPB diameter of $d_{SPB}=$ 2 µm was injected into the microfluidic device and the SPB transport was observed via an optical microscope set-up.

For the rectangular pulse shape, the average steady state velocity remains about the same (Table 1) independent on the length of the pulse period. This was expected for the case that the magnetic field alteration rate is fast in comparison to the SPB steady state velocity (see Figure 13). The average steady state velocity is in the range of 100 µm/s; therefore, the SPBs will traverse a 5 µm wide domain within a timeframe of 20 ms. For the sinusoidal pulse shape, it is evident from Table 1 that the average steady state SPB velocity decreases with increasing pulse length. The decrease of the SPB velocity is attributed to the different switching times of the sinusoidal external magnetic field sequence if the cycle duration T is changed.

**Table 1:** Experimentally determined steady state velocities of 2 µm diameter SPBs $v_{SPB}$ for the two pulse shapes displayed in Figure 15 as a function of the pulse length. For the sinusoidal pulse shape, the experimentally determined average SPB acceleration $a_{SPB}$ within a pulse is also given.

	Rectangular	Sinusoidal	Sinusoidal
T/ms	$v_{SPB}\pm \Delta v_{SPB}$/µm/s	$v_{SPB}\pm \Delta v_{SPB}$/µm/s	$a_{SPB}\pm \Delta a_{SPB}$/µm/s^2^
120	126 ± 16	115 ± 30	13.1 ± 3.8
140	111 ± 24	142 ± 39	12.7 ± 3.4
160	79 ± 13	113 ± 31	12.4 ± 3.0
180	85 ± 23	110 ± 31	9.2 ± 2.0
200	106 ± 29	104 ± 30	3.7 ± 0.7
400	110 ± 31	96 ± 27	3.9 ± 0.8
600	73 ± 18	68 ± 18	2.7 ± 0.6
800	111 ± 30	64 ± 17	2.8 ± 1.2

According to Figure 14, where the SPB velocity is shown as a function of the magnitude of the external magnetic field’s z-component, the SPB velocity is increased with increasing $H_{ex,z}$. As for the sinusoidal pulse scheme the magnetic field alteration rate increases with decreasing switching time $\Delta t_{s}$ the corresponding acceleration acting on the SPBs is increased, too. For the short sinusoidal pulse period the rise to maximum field value occurs within about 30 ms (which corresponds to field switching within 60 ms), whereas for the longest pulses this rise occurs within 200 ms (corresponding to field switching within 400 ms). Therefore, during the motion of the particles from a position above one domain wall to a position above the adjacent domain wall, the average value of the external magnetic field and, hence, the average magnetic force acting on the SPB motion also decreases with increasing pulse length. This result is supported by the measured decreasing acceleration of the SPBs (Table 1), which was examined from the experimentally observed distance-time-diagram of the SPB motion along the x-axis, which, in contrast to the experiments with rectangular pulse shapes, revealed a quadratic increase of the SPB distance as a function of time if the sign of the external magnetic fields’ z-component is inversed. This finding is qualitatively manifested by the rounded corners within the distance-time-diagram of the SPB motion which is otherwise almost step-like in case of the minimal time resolution of 500 µs accessible in the experimental set-up. As a consequence for applications it is important to realize fast magnetic field switching by the external magnets, preferably by air coils. Moreover, the change of the SPB velocity as a function of the external magnetic field’s frequency for the sinusoidal pulse shape is in accordance to previous results [41,43], where the SPB velocity linearly increases as a function of the external magnetic field’s frequency up to the critical frequency [41,43]. As opposed to the sinusoidal pulse scheme, for the rectangular pulses where the switching time of the magnetic field pulses is almost negligible in comparison to the plateau time, the SPB steady state velocity is independent of the frequency of the external magnetic field. Hence, different pulse shapes might be adequate for the directed transport of SPBs for different applications.

## 5. Summary and Outlook

In conclusion, the present report described fundamentals and technology for a SPB transport based technology platform to be used for LOC or µTAS devices in biotechnology. This platform has a large potential for applications in biosensors as different tasks can be carried out by the SPBs on-chip.

The application of SPBs for diffusion enhancement has already been shown in separate work [106,107]. This task is particularly important for devices employing SPBs moving essentially in one plane close to a surface as it is necessary for techniques employing magnetic field landscapes emerging from a substrate. If in a µTAS or LOC device capture molecule functionalized SPBs are used, the analyte molecules have to find their way to the capture molecules. In all transport concepts using MFLs overlaid with dynamically varying external magnetic field pulses the SPBs are moving essentially in a plane close to the substrate surface. For high analyte molecule concentrations this is not a severe problem as there are enough analyte molecules in the volume around the SPB transport plane close to the SPBs. For small analyte molecule concentrations and large container or microfluidic channel heights, however, it is not straightforward that analyte molecules may find the capture molecule on the surface of the SPB. In the small containers of LOC or µTAS devices, the Reynolds number is small, resulting in laminar flows and in an increasing fraction of the fluid volume adhering to the walls. Conventional stirring which needs turbulent flow is therefore not efficient. The transport of an analyte molecule to a capture molecule immobilized on the surface of the SPB, therefore, is governed by slow molecular diffusion. As has already been shown, efficient active mixing by increasing the contact area between fluid lamellae due to the motion of the SPBs close to a surface even in containers with 250 times the height of the SPB diameter increases diffusion by about 1 order of magnitude [106,107].

The second task which can be solved by the capture molecule functionalized SPBs is collecting analyte molecules, separating them from other similar molecules causing interfering detection signals, and concentrating them in the detection area. In this way the density of the detected physical quantity and the signal-to-noise ratio are increased and the detection threshold of a given analysis system can be reduced accordingly.

Furthermore, a fast transport of SPBs at distances of several hundred nanometers above the topographically flat surface of the magnetically stripe patterned EB layer system while applying external magnetic field strengths of only a few millitesla enables the integration of state-of-the-art sensor concepts, e.g., surface plasmon resonance or magnetoresistive sensors, into this technology platform.

Due to the acceleration characteristics of the moving particles, magnetophoresis is also at hand, which may be used to separate particles with different hydrodynamic radii or particles with different loads.

The most important advantage of this technology platform, finally, is the possibility to transport many individual particles at the same time in parallel rows without agglomeration. This characteristic may enable the detection of several biomarkers simultaneously on the same chip, when particles functionalized with different capture molecules are used on the same chip.

The presented technology platform is now well understood quantitatively. It is a versatile tool expected to be implemented in real devices within the next few years.
